# An unusual Toll/MyD88-mediated *Drosophila* host defence against *Talaromyces marneffei*

**DOI:** 10.1080/19336934.2024.2398300

**Published:** 2024-09-06

**Authors:** Xiaoyue Wang, Qinglin Qu, Zi Li, Sha Lu, Dominique Ferrandon, Liyan Xi

**Affiliations:** a Dermatology hospital, Southern Medical University, Guangzhou, China; b Department of Clinical Laboratory, Zhuhai People’s Hospital, Zhuhai Clinical Medical College of Jinan University, Zhuhai, China; c Sino-French Hoffmann Institute, Guangzhou Medical University, Guangzhou, China; d Department of Dermatology, Sun Yat-sen Memorial Hospital, Sun Yat-sen University, Guangzhou, China; e Université de Strasbourg, UPR 9022 du CNRS, Strasbourg, France

**Keywords:** *Drosophila melanogaster* infection model, resistance, detection of fungal infections, talaromycosis, Toll pathway activation

## Abstract

Talaromycosis, caused by *Talaromyces marneffei* (*T. marneffei*, formerly known as *Penicillium marneffei*), is an opportunistic invasive mycosis endemic in tropical and subtropical areas of Asia with high mortality rate. Despite various infection models established to study the immunological interaction between *T. marneffei* and the host, the pathogenicity of this fungus is not yet fully understood. So far, *Drosophila melanogaster*, a well-established genetic model organism to study innate immunity, has not been used in related research on *T. marneffei*. In this study, we provide the initial characterization of a systemic infection model of *T. marneffei* in the *D. melanogaster* host. Survival curves and fungal loads were tested as well as Toll pathway activation was quantified by RT-qPCR of several antimicrobial peptide (AMP) genes including *Drosomycin*, *Metchnikowin*, and *Bomanin Short 1*. We discovered that whereas most wild-type flies were able to overcome the infection, *MyD88* or *Toll* mutant flies failed to prevent fungal dissemination and proliferation and ultimately succumbed to this challenge. Unexpectedly, the induction of classical Toll pathway activation readouts, *Drosomycin* and *Bomanin Short 1*, by live or killed *T. marneffei* was quite limited in wild-type flies, suggesting that the fungus largely escapes detection by the systemic immune system. This unusual situation of a poor systemic activation of the Toll pathway and a strong susceptibility phenotype of *MyD88*/*Toll* might be accounted for by a requirement for this host defence in only specific tissues, a hypothesis that remains to be rigorously tested.

## Introduction

Talaromycosis, an opportunistic infectious disease caused by *Talaromyces marneffei* (*T. marneffei*, formerly known as *Penicillium marneffei*) is mainly endemic to Southeast Asia and some southern regions of China. Talaromycosis usually occurs in immunocompromised individuals, such as those with acquired immunodeficiency syndrome (AIDS), and causes fatal complications. The number of talaromycosis cases has been increasing over recent years, with rising reports of this disease occurring in HIV-negative individuals [1,2]. In 2021, scientists from nations where talaromycosis is endemic raised a global call for it to be recognized as a neglected tropical disease [3]. In addition, with global travel increasing, talaromycosis has spread far beyond the original epidemic region [4].

*T. marneffei* is a thermal dimorphic fungus. It grows in a filamentous hyphal form that can produce asexual spores (conidia) at 25°C. The velvety colonies are yellow-grey or green-grey with a brick-red water-soluble pigment that diffuses in the medium. At 37°C, *T. marneffei* grows in an uninucleate yeast form that divides by fission. The colonies are yeast-like and whitish without visible pigment. It is generally believed that patients inhale pathogenic conidia which can adhere to the host extracellular matrix and to the bronchoalveolar epithelium. Then, conidia are phagocytosed by pulmonary macrophages and neutrophils. Once internalized, they rapidly transition to the yeast form [5] and evade the killing by several strategies, such as producing catalase- peroxidase [6,7] and exploiting alternative carbon sources [8–10]. Finally, *T. marneffei* replicates inside phagosomes and escape into the cytoplasmic environment with the rupture of the phagosomal vacuoles [11,12].

The pathogenicity of *T. marneffei* is not fully understood yet. A number of experimental infection models have been established to study the immunological interaction between *T. marneffei* and the host, including *Caenorhabditis elegans* [13], *Galleria mellonella* [14,15], *Danio rerio* [16,17], *Mus musculus* [18] and macrophage cell lines [12]. *Drosophila melanogaster* is a well-established genetic model organism to study innate immunity [19–21]. It is arguably the invertebrate species in which the host defence against fungal infections is best understood thanks to genetic analysis and the possibility to alter the expression of any gene in a time-controlled and tissue/cell type-specific manner [22–38]. A major host defence against systemic fungal infections and most Gram-positive bacterial infections is mediated by the Toll pathway [22] whereas a second NF-κB pathway, Immune deficiency (IMD) is required for protection against Gram-negative bacterial infections. Fungal infections are either sensed by the circulating ß-(1-3)-glucan sensor GNBP3 or by the detection of pathogenic protease activity [23–26]. Once triggered, host proteolytic cascades lead, on the one hand, to the cleavage of pro-Spätzle into an active cytokine ligand of the Toll membrane receptor and on the other to the activation of a protostome-specific defence, melanization. Once activated, Toll triggers a NF-κB type signal transduction pathway that leads to the induction of the transcription of Toll effector genes. These include genes encoding potent antimicrobial peptide (AMP) genes such as *Drosomycin* or *Metchnikowin*. Importantly, the action of the Toll pathway against many fungal or Gram-positive bacteria appears to be mediated by Bomanin (Bom)-encoding genes, 10 of which are clustered at the 55C locus [33]. This locus mediates not only the resistance against infections, but some Bomanins also protect against the action of secreted mycotoxins [31]. The promoters of *BomS1* and *Drosomycin* contains NF-κB response elements that are optimal for binding the Dorsal and Dorsal-related Immune Factor (DIF) transcription factors [39].

In this article, we have used the fruit fly *D. melanogaster* to establish an infection model of *T. marneffei*. We report that *MyD88* and *Toll* are required to prevent the proliferation of injected *T. marneffei* conidia, even though this opportunistic pathogen is a poor elicitor of this pathway.

## Materials and methods

### Microbial strains

*Talaromyces marneffei* strain SUMS0152 was isolated from the bone marrow culture of a patient diagnosed with a *T. marneffei* infection. This strain was identified as *T. marneffei* by its morphology and Internal Transcribed Spacer (ITS) rRNA sequencing (Accession No. AB353913.1). The conidia were harvested in PBS containing 0.01% Tween-20 (PBST) after 10–14 days of culture on potato dextrose agar (PDA) medium at 25°C. The conidial suspension was purified by filters to eliminate hyphae and other impurities and cell number was determined with a haemocytometer.

*Micrococcus luteus* CGMCC#1.2299 was cultured in Luria‐Bertani (LB) at 37°C for 24 h. The bacteria were then washed twice using PBS and concentrated to OD_600_ = 50 for injection.

### Fly strains

Fly lines were raised on standard food at 25°C with 65% humidity. For 1 L of standard food medium, 77.7 g cornmeal, 63.2 g glucose, 32.19 g yeast, 31.62 g sucrose, 1.5 g nipagin were diluted into 15 mL ethanol, 9 g agar, 0.726 g calcium chloride, 2 g potassium sorbate, and distilled water were used. For the food medium without potassium sorbate, potassium sorbate was not added to the preparation. For 1 L food medium with gentamicin, 0.05 g gentamicin was added into the standard food.

*w*^*1118*^ [*A5001*] flies were used as wild‐type control [40]. *MyD88*^*c03881*^ mutant flies have been generated in the *w*^*1118*^ [*A5001*] background [40]. *Toll* mutant flies were obtained by crossing the heterozygous mutants, *Toll*^*632*^ and *Toll*^*9QRE*^, at 25°C. *ΔAMP14* mutant flies lack most AMP gene families, a kind gift of Bruno Lemaitre [41].

### Drosophila infection

For infection, 20–25 female flies aged 5–7 days were put into a tube, and each single experiment contained one tube for the blank control group, one tube for the negative control group and three tubes for the experimental group.

In the injection model, 4.6nL microbial suspension was injected into the thorax of the flies using a microcapillary connected to a Nanoject III (Drummond). The same volume of PBST was injected for the negative control.

In the natural infection model, flies were transferred into a 50 ml centrifuge tube that contained 5 mL microbial suspension, and were shaken gently for 30 s. The flies were then put on a filter under a vacuum to dry them out. The same volume of distilled water was used for the negative control.

### Survival tests

Once infected, the flies were transferred to the food without potassium sorbate and raised at 29°C unless otherwise indicated. Flies that died within 2 h after infection were not taken into account as they are likely killed by the trauma of the injection procedure. Surviving flies were counted every day until the 14th day post infection. Flies were transferred to new tubes every 3 days. The data shown correspond to pooled data.

### Quantification of the fungal load

For fungal burden, single flies were put each into a 200 μL tube containing a 2.0-mm grinding zirconium bead in 100 μL PBST. The flies were then homogenized by using a Mixer Mill 400 (Retsch) at a frequency of 30/min for 30 s. The tissue homogenate was plated onto PDA plates supplemented with antibiotics. These plates were sealed with parafilm and cultured at 25°C. Colony forming units (CFUs) were counted after 72 h. For fungal load upon death (FLUD), dying flies were collected every half hour after flies began to die and CFU counts were then made.

### UV-killed and heat-killed T. marneffei

For the preparation of ultra violet-killed (UV-killed) *T. marneffei*, the conidial suspension was plated on PDA plates, and then exposed to the UV-light for 3 h. The plates were enclosed with parafilm and cultured at 25°C. After 72 h, the plates without any colony were sorted and the dead conidia were resuspended in PBST to measure the concentration.

For the preparation of heat-killed *T. marneffei*, the conidial suspension was heated at 100°C for 30 min.

### Antimicrobial peptide genes expression measurement

Steady-state expression levels of *Drosomycin (Drs)*, *Metchnikowin (Mtk)*, *Bomanin Short 1 (BomS1)*, and *Daisho* genes was measured by RT-qPCR. Five anesthetized flies were collected into a tube and crushed to extract RNA using RNAiso Plus (Takara). The RNA samples were then reverse transcribed into cDNA using a kit (R323–01, Vazyme). After that, cDNA samples were diluted tenfold and used to run qPCR according to the protocol provided by the reagent company (Q311–02, Vazyme). The results were normalized by the counts of *RpL32* reference gene using the ΔΔCt method. The sequences of primers (synthesized by Sangon Biotech) are shown in Table 1. The amplification efficiency is similar between AMP gene primers and *Rpl32* primers.

**Table 1.** Primers used in this work.

Primer		Sequence
RpL32	FW	GACGCTTCAAGGGACAGTATCTG
RpL32	RV	AAACGCGGTTCTGCATGAG
Drosomycin	FW	CGTGAGAACCTTTTCCAATATGATG
Drosomycin	RV	TCCCAGGACCACCAGCAT
Metchnikowin	FW	CGTCACCAGGGACCCATTT
Metchnikowin	RV	CCGGTCTTGGTTGGTTAGGA
BomaninS1	FW	CAATGCTGTTCCACTGTCGC
BomaninS1	RV	CGTGGACATTGCACACCCTG
Daisho1	FW	TCTCTTGGCCATGTTCGCT
Daisho1	RV	TACTGGGTGTTGTCGGTCTG
Daisho2	FW	TGCGGCTTTTTCTTCGCTCT
Daisho2	RV	TGTGTCCGCCAGCATGAAT

### Statistical analysis

Unless otherwise stated, most experiments have been performed at least thrice. Statistical analysis was performed using GraphPad Prism 7. The Shapiro– Wilk normality test was used to analyse normal distribution. Mann–Whitney test and Kruskal–Wallis test together with Dunn’s multiple comparisons post-hoc test were used to analyse fungal loads and RT-qPCR. The log‐rank test was used to analyse survival experiments.

## Results

### T. marneffei injection killed immunodeficient MyD88 mutant flies

**Figure 1.**
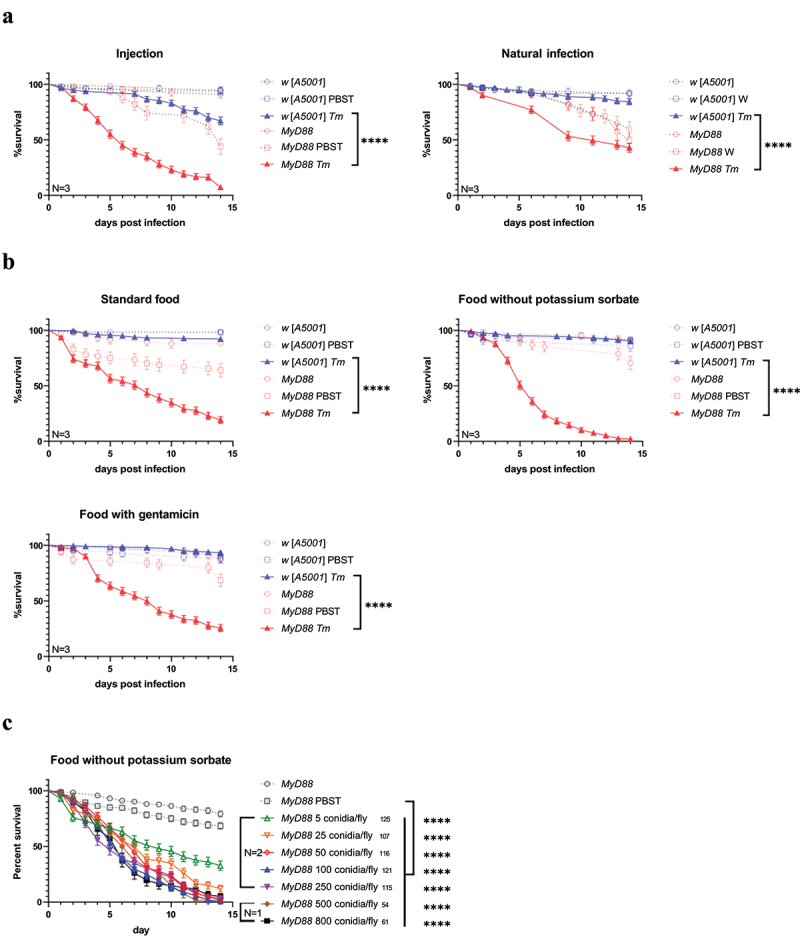
Susceptibility of *MyD88* mutant flies to *talaromyces marneffei* infection.(a) injection model and natural infection model of *talaromyces marneffei* in *w* [*A5001*] flies and *MyD88* mutant flies at the dose of 100 conidia/fly and 10^6^ conidia/mL (5 mL for each tube of flies), respectively. (b) Standard food, food without potassium sorbate and food with gentamicin were used in the injection model at the dose of 100 conidia/fly. (c) Dose–response curves of injected *MyD88* mutant flies fed on the food without potassium sorbate. The quantity of flies in total is indicated to the right of the group.

We first tried to construct a *T. marneffei* infection model of *Drosophila melanogaster* using two common methods of infection, namely septic injury (injection) and natural infection. Neither of the two infection routes led to a consistent demise of wild-type (*w*^*1118*^ [*A5001*]) flies upon exposure to *T. marneffei* conidia (Figure 1a; see also Figure 1b for wild-type hosts). Considering the essential role of Toll pathway in *Drosophila melanogaster*’s host defence against Gram-positive bacteria and fungi, we then tested *MyD88* immunodeficient mutant flies, in which Toll pathway signalling is blocked, to establish a *T. marneffei* infection model. Natural infection did not significantly kill *T. marneffei* conidia-injected *MyD88* mutant flies faster than noninjected or PBS-injected controls, likely because of the barrier of the insect cuticle. In contrast, the septic injury breaks through the exoskeleton barrier and injected conidia caused the death of *MyD88* flies. The survival curves of the *MyD88* mutant flies injected with *T. marneffei* displayed a clearcut phenotype, in which case nearly all of the flies finally succumbed to *T. marneffei* on the 14th day post infection (dpi).

The data correspond to pooled data from three independent experiments, unless otherwise indicated. N, times of independent experiments; *Tm*, *Talaromyces marneffei* infection; W, water treatment. ****, *P*<0.0001.

The composition of the food medium in terms of added preservatives also impacted the survival rates of the *MyD88* mutant flies (Figure 1b). The addition of potassium sorbate to the food medium reduced the killing rate, suggesting that it affects the virulence of the injected fungus. The addition of gentamicin to the food did not alter the mortality rate, suggesting that the microbiota did not provide a major contribution to the death of *T. marneffei*-infected *MyD88* flies. Please, note that PBS-injected *MyD88* flies occasionally display a mild sensitivity to this challenge that is alleviated by gentamicin treatment. Hence, we used food medium without potassium sorbate for further experiments.

To optimize the injection dose, the conidial suspension was diluted to different concentrations. *T. marneffei* was able to kill about 70% of *MyD88* mutant flies with as low a dose as 5 conidia/fly (Figure 1c). All immunodeficient flies became more sensitive to infection when higher concentrations were used. However, there was no apparent difference among the groups of 100, 250, 500 and 800 conidia/fly. We chose to use 100 conidia/fly as a standard concentration in subsequent experiments.

### The toll but not the IMD pathway appears to be required for protection of the host against T. marneffei

**Figure 2.**
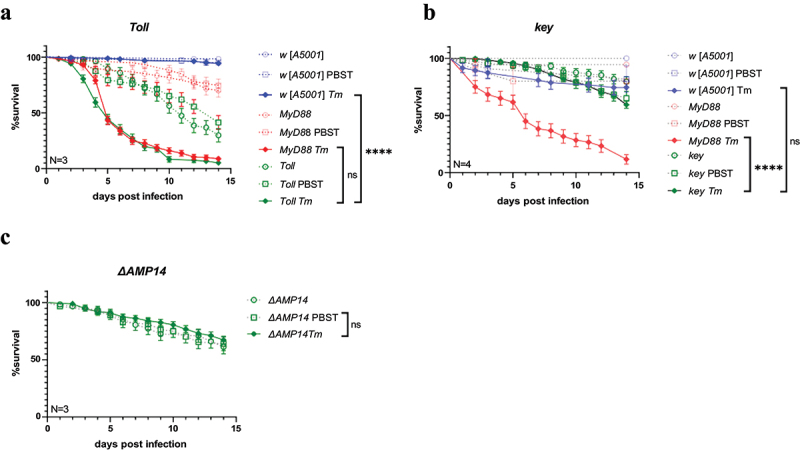
Susceptibility of *Toll*, *key*, and *ΔAMP14* mutant flies to *talaromyces marneffei* infection.(a) survival of *Toll* mutant flies injected with *T. marneffei* at the dose of 100 conidia/fly. (b) Survival of *key* mutant flies injected with *T. marneffei* at the dose of 100 conidia/fly. (c) Survival of *ΔAMP14* mutant flies injected with *T. marneffei* at the dose of 100 conidia/fly. The data correspond to pooled data from at least three independent experiments. N, times of independent experiments; *Tm*, *talaromyces marneffei* infection. ****, *P*<0.0001; ns, no significance, *P*>0.05.

Even though the *MyD88* allele we have tested has been extensively characterized in a previous study [31], a remote possibility remains that the phenotype we observe is due to a second-site mutation that would specifically impact the sensitivity to *T. marneffei* and not to other fungal infections. We therefore chose to test a mutant that affects the gene that encodes the Toll receptor itself. As shown in Figure 2a, *Toll* mutants were as sensitive as *MyD88* mutants to *T. marneffei*. In contrast, flies mutant for the canonical IMD pathway mutant *kenny* (*key*) resisted as well as wild-type flies (Figure 2b). In keeping with this latter result and a previous study [41], flies lacking most of the AMP gene families did not exhibit any sensitivity to *T. marneffei* (Figure 2c). These flies lack the classical antifungal AMPs Drosomycin and Metchnikowin and are generally not highly susceptible to fungal infections [41].

### T.Marneffei proliferates in MyD88 mutant flies

**Figure 3.**
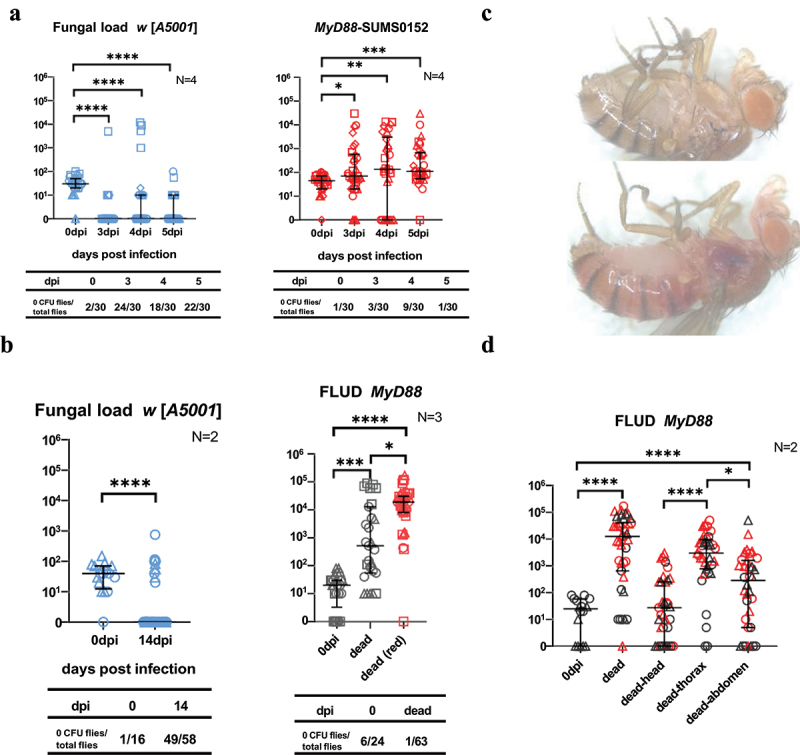
Proliferation and dissemination of *Talaromyces marneffei* in *MyD88* flies.(a) fungal loads of *w*[*A5001*] flies and *MyD88* mutant flies on 3-5 days post infection (dpi). (b) Fungal load of *w* [*A5001*] flies on 14 dpi and fungal load upon death (FLUD) of *MyD88* mutant flies. (c) Different appearances of *MyD88* mutant flies’ carcasses under the stereomicroscope at 80 × magnification. (d) Fungal loads of different tagmata (head, thorax, abdomen) of dying *MyD88* mutant flies. (b, d) red data points correspond to dead flies that present a reddish color, as shown in (c). Flies were infected at the dose of 100 conidia/fly. The data correspond to pooled data from several independent experiments (results from each single experiment are represented by a specific symbol shape (circles, triangles, squares, and diamonds)) and described by Median with interquartile range for they were non-normally distributed data. N, times of independent experiments. *, *P* < 0.05; **, *P* < 0.01; ***, *P* < 0.001; ****, *P* < 0.0001; ns, no significance, *P* > 0.05.

As shown by survival curves (Figure 1), *MyD88* mutant flies began to die on the third day post-infection (dpi), and almost half of them would succumb to *T. marneffei* injection in the first 5 days. Therefore, fungal loads were measured on the 3–5 dpi period to explore if there was a connection between fly death and fungal proliferation. The fungal loads of the single *MyD88* mutant flies significantly increased after infection, as compared to the decreased fungal loads observed in most *w* [*A5001*] flies (Figure 3a). Thus, *MyD88* plays a role in the resistance against *T. marneffei* proliferation. In contrast to a *Candida glabrata* [30] or *Aspergillus fumigatus* [31] challenge, *T. marneffei* appeared to be cleared in a vast majority of flies (Figure 3a-b).

Fungal load upon death (FLUD) is a detection index of the upper limit of fungal load in single flies. The FLUD of infected *MyD88* mutant flies was higher than the initial injection dose, though it strikingly did vary over a four log concentration range (Figure 3b). Thus, whereas an increased fungal burden does contribute to the demise of each fly, it may not be the only factor leading to the death of the infected individual. Most wild-type control flies had cleared the infection when they were checked, when still alive, at 14 days post-infection. We noted that some carcasses of the dead *MyD88* flies turned red (Figure 3c), possibly as a result of the production and secretion of *T. marneffei*’s secondary metabolites, such as red pigments [42]. Thus, we marked the data of FLUD into two groups according to the different appearances of the cadavers and found in a preliminary experiment that the flies with red carcasses usually possessed higher fungal loads (Figure 3b). This trend was, however, not confirmed in subsequent experiments in which we assessed in dying flies whether the fungus had disseminated to other tagmata than the thorax. Consistent with the injection taking place in the thorax, the highest burden was measured in the thorax, followed by the abdomen and head (Figure 3d). We conclude that *MyD88* function contributes to preventing the proliferation and dissemination of the fungus inside flies.

### The antifungal peptide genes were only mildly induced by T. marneffei infection

**Figure 4.**
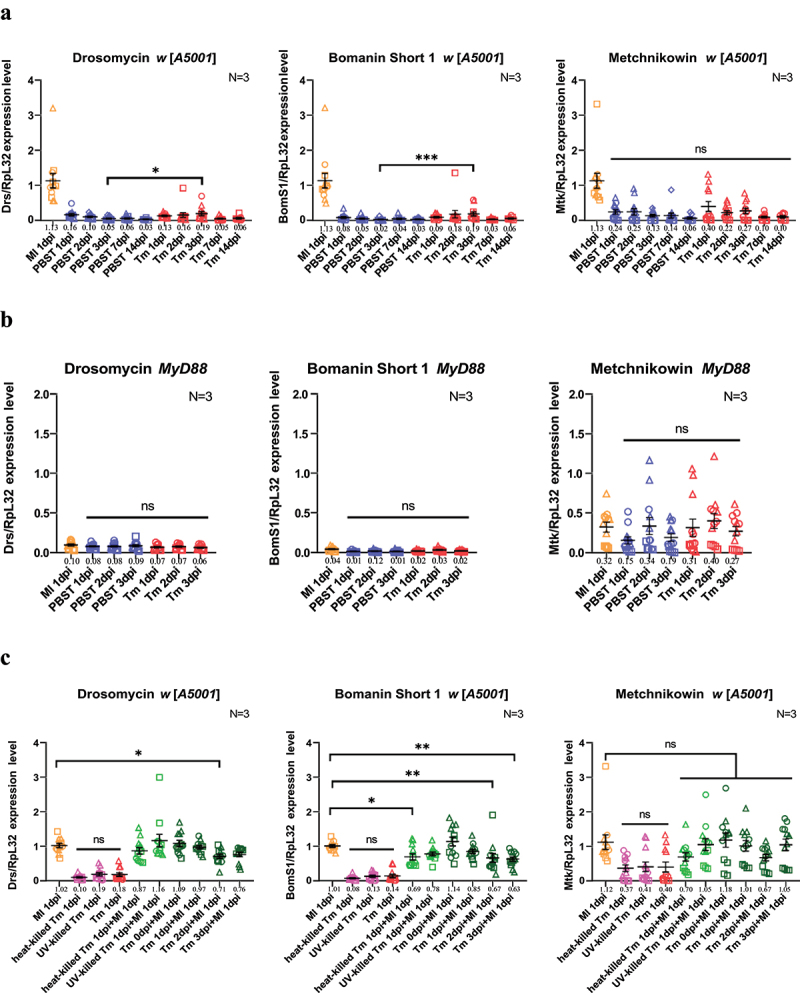
*Talaromyces marneffei* is a poor elicitor of the toll pathway.(a) steady-state transcript levels of *Drosomycin*, *BomS1* and *Metchnikowin* on 1-3 dpi, 7dpi, and 14dpi flies as measured by RTqPCR in wild-type *w* [*A5001*] flies. (b) Steady-state transcript levels of *Drosomycin*, *BomS1* and *Metchnikowin* on 1-3 dpi, 7dpi, and 14dpi flies as measured by RTqPCR in *MyD88* mutant flies. (c) Steady-state transcript levels of *Drosomycin*, *BomS1* and *Metchnikowin* in *w* [*A5001*] flies infected with *T. marneffei* (live conidia, unless otherwise indicated: UV- or heat-killed) and/or *Micrococcus luteus* (OD_600_ = 50, 4.6 nL). In the case of double infections, flies were first challenged with *T. marneffei* and then secondarily after 0 to 3 days (tm *x*dpi) as indicated with *M. luteus* (the time of analysis was one day after *M. luteus* challenge). (A-C) flies were infected at the dose of 100 conidia/fly.

Since the *MyD88* mutant flies easily succumbed to *T. marneffei* injection, it seemed likely that the antifungal peptides regulated by the Toll/MyD88 pathway might be involved in Drosophila’s resistance against *T. marneffei*. It is well known that the *Drosomycin* and *Metchnikowin* genes encode two important antimicrobial peptides (AMPs) active against filamentous fungi [20]. Short-form Bomanins (Boms), a set of novel immune secreted peptides, appear to be essential effectors of the Toll pathway [36]. Here, we tested the steady-state transcript levels of *Drosomycin* (*Drs*), *Metchnikowin* (*Mtk*), *Bomanin Short 1* (*BomS1*), and *Daisho1/Daisho2* genes using RT-qPCR. Unexpectedly, *T. marneffei* only mildly induced *Drs* and *BomS1* mRNA expression in the *w* [*A5001*] flies (Figure 4a; Fig. S1) as compared to the response induced by the injection of the Gram-positive bacterium *Micrococcus luteus*. Indeed, the levels of induction of those genes were not significantly different from those measured in PBS-injected control flies except on day 3. As expected, no induction of these peptide genes was observed in *MyD88* mutant flies (Figure 4b). Even though *Mtk* is expressed at somewhat higher levels, this may not reflect the activation of the Toll pathway since similar levels of expression were observed in *MyD88* flies. Indeed, *Mtk* has been shown to be also regulated by the IMD pathway [43].

Each panel represents the pooled data from three independent experiments, each with four biological replicates of samples of five flies. Mean with SEM are displayed for each condition, and the means are indicated beneath the x-axis. Tm, *Talaromyces marneffei*; Ml, *Micrococcus luteus*. N, times of independent experiments. Results from each single experiment are represented by a specific symbol shape (circles, triangles, squares, and diamonds). *, *P* < 0.05; **, *P* < 0.01; ***, *P* < 0.001; ns, no significance, *P* > 0.05.

The low level of induction of the systemic Toll pathway response may result from a failure to detect the infection or from an active repression of its activation by the invading fungus. To test the latter possibility, we designed a co-infection experiment to explore if *T. marneffei* can suppress *Drs* or *BomS1* expression. The Gram-positive bacteria *M. luteus* was used to stimulate the Toll pathway following the prior injection of *T. marneffei* up to 3 days earlier, to determine if the steady-state transcript levels of *Drs* or *BomS1* would be affected. Data showed that the injection of *T. marneffei* 2 or 3 days in advance significantly decreased the transcription level of *Drs* and *BomS1* induced by *M. luteus*, but the effect appeared very mild and was not confirmed upon the injection of 1,000 conidia instead of 100 (Figure 4c, Fig. S2). In addition, there was no influence of a *T. marneffei* pre-challenge on the expression of *Mtk* induced by *M. luteus* at the two doses tested (Figure 4c, Fig. S2). We also injected heat-killed or UV-killed conidia to assess whether the ß-(1-3)-glucans of the *T. marneffei* cell wall can be sensed by the fly immune system. Killed conidia failed to detectably induce the expression of *Drs*, *BomS1* or *Mtk* at day 1, like live conidia. Of note, the injection of killed conidia failed to cause the demise of wild-type or *MyD88* flies (Fig. S3).

## Discussion

In this study, we report the preliminary characterization of a *T. marneffei* systemic infection model in the *D. melanogaster* host in which conidia are directly injected in the haemocoel at the level of the thorax. Only *MyD88* and *Toll* mutant flies succumb to this challenge whereas most wild-type flies appear to clear the infection at the low dose used in our experiments. Since the fungal burden is increasing and that the fungus disseminates throughout the body of the immuno-deficient host, it follows that *MyD88* is required for resistance to this fungus.

Whereas *Aspergillus fumigatus* hardly induces *Drosomycin* expression [31], it nevertheless elicits enhanced levels of short *Bomanin* steady-state transcripts that can be detected by RTqPCR. *T. marneffei* also appears to be a poor elicitor of the Toll pathway as judged from the very limited induction of classical Toll pathway activation readouts, *Drosomycin* and *BomS1*, by either alive or killed *T. marneffei* for a period ranging from 1 to 14 days. This observation first raises the question whether the cell wall component detected by GNBP3, ß-(1,3)-glucans [23], is readily accessible to the sensor in conidia. In the pathogenic yeast *Candida albicans*, the yeast and not the filamentous form of the fungus can be bound by the ß-(1,3)-glucan receptor dectin-1 and this happens only at the budding scar [44]. One possibility is therefore that the injected conidia rapidly form hyphae and not yeasts upon injection into the host and would thereby avoid detection by the circulating GNBP3 ß-(1,3)-glucan sensor. Furthermore, our data also suggests that the fungus does not secrete proteases that would be detected by the Persephone arm of Spätzle maturation [23–25].

In wild-type flies, we have a somewhat paradoxical situation. Even though the fungus induces at best a mild expression of *Drosomycin* and *BomS1* only 3 days after the injection of conidia and no detectable induction of currently known AMPs with potential antifungal activity (*Daisho1/Daisho2*, other *Bomanin* genes (Figs. S1 &amp;S4)), the fungus is nevertheless controlled in a Toll-dependent manner in the wild-type. It will be thus needed to investigate earlier time points to exclude the possibility of a short-lived rapid induction of the pathway, which has never been documented before in the case of the Toll pathway as most of its regulated genes are expressed with a relatively slow kinetics as exemplified by the expression of *Drosomycin* [29,45]. Genetically, we should also investigate mutant lines that are lacking all 55C Bomanins. Whereas Bomanins have been shown to play an important role in the protection against *A. fumigatus* mycotoxins [31], they have also been reported to be involved in the resistance against fungal or bacterial pathogens [33,36].

An alternative explanation to account for the discrepancy between the poor induction of Toll pathway target genes and the sensitivity of *Toll* or *MyD88* mutants is that the Toll pathway gets activated in only a specific tissue in which it is critically required for protection against *T. marneffei*. We note that this fungus has been detected, rarely, in the cerebrospinal fluid of patients [46]. It will be thus important to silence Toll pathway components in specific tissues, including the blood brain barrier.

Finally, while our work clearly supports a role for *Toll* and *MyD88* being required in resistance against *T. marneffei* infection, we do not exclude a role also in resilience against infection and secreted mycotoxins given the puzzlingly highly variable fungal load upon death over several logs we have measured in recently killed flies (Figure 3 b-d). Indeed, some *MyD88* flies appear to have succumbed to only a very low fungal burden; as documented in the case of *A. fumigatus* [31], these immune-deficient flies may have died of exposure to secreted virulence factors that are normally counteracted by host defence in wild-type flies. Alternatively, it might reflect different morphologies of the fungus *in vivo*, possibly with various ratios of yeast to filamentous forms occurring in different host flies.

## Supplementary Material

T_marneffei_Supplemental Figures_Finals.docx

## Data Availability

The raw data supporting the findings of this study have been deposited in the Figshare repository and are accessible at: DOI: 10.6084/m9.figshare.26489167.
