# Supplementary material for: An unusual Toll/MyD88-mediated Drosophila host defence against Talaromyces marneffei
Source: Fly (Austin). 2024 Sep 6;18(1):2398300. doi: 10.1080/19336934.2024.2398300 (PMC11382710; doi:10.1080/19336934.2024.2398300)
Supplement: T_marneffei_Supplemental Figures_Finals.docx [file KFLY_A_2398300_SM7143.docx]

Supplemental Materials to:

“An unusual Toll/MyD88-mediated *Drosophila* host defense against *Talaromyces marneffei*?”

Fig. S1 Transcript levels of *Daisho1/Daisho2* in the *w* [*A5001*] flies.

Fig. S2 Transcript levels of *Drs*, *BomS1*, and *Mtk* in the *w* [*A5001*] flies at the high infection dose.

Fig. S3 Survival of *MyD88* mutant flies injected with UV-/heat-killed *Talaromyces marneffei*.

Fig. S4 Transcript levels of *Bom* genes in *w* [*A5001*] flies.

**Daisho1 *w* [*A5001*]**

**Daisho1/RpL32 expression level**

**4** N=2


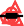

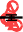

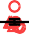

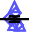

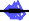

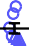

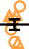


ns

**Daisho2 *w* [*A5001*]**

**4** N=2


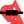

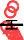

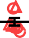

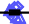

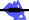

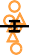


ns

**Daisho2/RpL32 expression level**

**3 3**

**2 2**

**1 1**

**0**

1.02 0.21 0.06 0.12 0.22 0.18 0.08

**0**

1.02 0.20 0.07 0.10 0.27 0.21 0.10

Fig. S1: Transcript levels of *Daisho1/Daisho2* in the *w* [*A5001*] flies.

The flies were injected with *T. marneffe****i*** at the dose of 100 conidia/fly. Each panel represents the pooled data from two independent experiments, each with four biological replicates of samples of five flies Mean with SEM are displayed for each condition, and the means are indicated beneath the x axis. The data was described by Mean with SEM. N, times of independent experiments; Tm, *Talaromyces marneffei* infection. ns, no significance, *P*>0.05.

**Drosomycin *w* [*A5001*]**

ns

**Drs/RpL32 expression level**

**5**


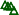

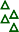

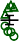

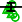


ns

**4**

**3**

**2**

**1**

N=2

**Bomanin Short 1 *w* [*A5001*]**

N=2

ns

**BomS1/RpL32 expression level**

**4**


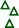

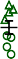

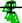

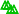


✱

**3**

**2**

**1**

**Metchnikowin *w* [*A5001*]**

N=2

ns

**Mtk/RpL32 expression level**

**8**


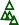

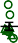

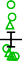


ns

**6**

**4**

**2**

**0**

1.07 0.22 0.30 0.28 1.90 0.97 1.18 0.84 2.21 1.42

**0**

1.05 0.08 0.15 0.37 1.27 0.54 1.10 0.92 1.71 1.12

**0**

1.06 0.42 0.61 1.04 2.35 1.69 1.41 1.05 2.73 1.83

Fig. S2: Transcript level of *Drs*, *BomS1*, and *Mtk* in the *w* [*A5001*] flies at the high infection dose.

The flies were injected with *T. marneffei* at the dose of 1000 conidia/fly. Each panel represents the pooled data from two independent experiments, each with four biological replicates of samples of five flies. Mean with SEM are displayed for each condition, and the means are indicated beneath the x axis. N, times of independent experiments; results from each single experiment are represented by a specific symbol shape (circles, triangles, squares, and diamonds). Tm, *Talaromyces marneffei* infection. *, *P*<0.05; ns, no significance, *P*>0.05.

***MyD88*-killed *Tm***

**100**

N=2

*MyD88*-PBST

*MyD88-*UV killed*Tm* ns

*MyD88-*heat killed*Tm*

**50**

**%survival**

**0**

**0 1 2 3 4 5**

**hours post infection**

**Fig. S3: Survival of *MyD88* mutant flies injected with UV-/heat-killed *Talaromyces marneffei*.**

Survival of *MyD88* mutant flies injected with UV- or heat-killed *T. marneffei* at the dose of 100 conidia/fly. The data correspond to pooled data from two independent experiments. N, times of independent experiments; *Tm*, *Talaromyces marneffei*. ns, no significance, *P*>0.05.

**BomS2 in *w* [*A5001*]**

ns N=2

**BomS2/RpL32 expression level**

**3**


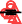

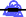

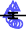

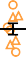


ns

ns

ns

**BomS3 in *w* [*A5001*]**

**3**


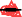

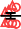

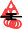

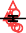

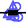

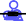

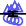

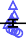

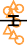


ns

ns

ns

ns

**BomS3/RpL32 expression level**

N=2

**BomS4 in *w* [*A5001*]**

ns N=2

**BomS4/RpL32 expression level**

**3**


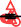

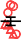

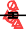

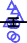

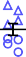


ns

ns

ns

**2 2 2**

**1 1 1**

**0 0 0**

**BomS5 in *w* [*A5001*]**

**BomS5/RpL32 expression level**

**3**


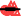

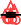

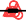

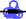

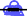

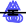

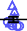

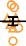


ns

✱

ns

ns

N=2

**BomS6 in *w* [*A5001*]**

ns N=2


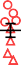

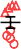

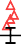

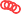

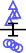

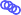

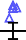

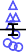


ns

ns

ns

**BomS6/RpL32 expression level**

**3**

**BomBc1 in *w* [*A5001*]**

N=2

**BomBc1/RpL32 expression level**

**3**


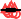

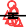

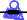

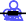

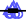

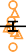


ns

✱

ns

ns

**2 2 2**

**1 1 1**

**0 0 0**

**BomBc2 in *w* [*A5001*]**

**BomBc2/RpL32 expression level**

N=2

**3**


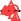

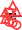

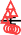

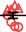

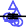

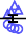

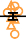


ns

ns

ns

ns

**BomT1 in *w* [*A5001*]**

**3** ns N=2


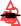

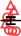

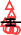

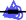

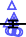

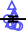


ns

ns

ns

**BomT1/RpL32 expression level**

**BomT2 in *w* [*A5001*]**

**3**


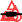

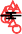

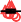

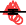

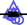

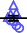

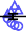

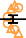


ns

✱

ns

ns

**BomT2/RpL32 expression level**

N=2

**2 2 2**

**1 1 1**

**0 0 0**

**Fig. S4: Transcript level of *Bom* genes in *w* [*A5001*] flies.**

The flies were injected with *T. marneffei* at the dose of 100 conidia/fly. Each panel represents the pooled data from two independent experiments, each with four biological replicates of samples of five flies Mean with SEM are displayed for each condition. N, times of independent experiments; results from each single experiment are represented by a specific symbol shape (circles, triangles, squares, and diamonds).Tm, *Talaromyces marneffei* infection. *, *P*<0.05; ns, no significance, *P*>0.05.
